# The Effect of Co-Verbal Remote Touch on Electrodermal Activity and Emotional Response in Dyadic Discourse

**DOI:** 10.3390/s21010168

**Published:** 2020-12-29

**Authors:** Angela Chan, Francis Quek, Haard Panchal, Joshua Howell, Takashi Yamauchi, Jinsil Hwaryoung Seo

**Affiliations:** 1 Department of Computer Science and Engineering, Texas A&M University, College Station, TX 77840, USA; quek@tamu.edu (F.Q.); haard.panchal@tamu.edu (H.P.); howjosh@tamu.edu (J.H.); 2 Department of Psychology, Texas A&M University, College Station, TX 77840, USA; takashi-yamauchi@tamu.edu; 3 Department of Visualization, Texas A&M University, College Station, TX 77840, USA; hwaryoung@tamu.edu

**Keywords:** remote touch, co-verbal remote touch, remote touch in video-teleconferencing, skin conductance response, electrodermal activity

## Abstract

This article explores the affective impact of remote touch when used in conjunction with video telecon. Committed couples were recruited to engage in semi-structured discussions after they watched a video clip that contained emotionally charged moments. They used paired touch input and output devices to send upper-arm squeezes to each other in real-time. Users were not told how to use the devices and were free to define the purpose of their use. We examined how remote touch was used and its impact on skin conductance and affective response. We observed 65 different touch intents, which were classified into broader categories. We employed a series of analyses within a framework of behavioral and experiential timescales. Our findings revealed that remote touches created a change in the overall psychological affective experience and skin conductance response. Only remote touches that were judged to be affective elicited significant changes in EDA measurements. Our study demonstrates the affective power of remote touch in video telecommunication, and that off-the-shelf wearable EDA sensing devices can detect such affective impacts. Our findings pave the way for new species of technologies with real-time feedback support for a range of communicative and special needs such as isolation, stress, and anxiety.

## 1. Introduction

Although communication through touch plays a significant role in natural human interaction, it is largely absent as a channel of remote communication alongside other modes such as video, voice, and text. There has been little research that explores the impact of the use of remote touch in multimodal communication, and whether and how it may impact affective experience as a communicative channel. To explore this area of research, we developed a system that allows dyads to touch one another during remote communication. By squeezing the input device, the other interlocutor receives a kinesthetic squeeze, generated by a constricting fabric armband device worn on their upper arm.

In this study, we recruited couples in committed relationships to participate in discussions with one another through a video tele-conference while separated into different rooms. Subjects were instructed to share their perspectives on emotionally charged topics based on videos they had just watched. The touch devices were given to them to use in one of two discussion sessions. Empatica’s E4 wristbands [1] were used to record electrodermal activity (EDA) for both participants, giving us a window into the instantaneous emotive experience with respect to discursive interaction.

This paper aims to investigate two main research questions: (1) How is remote touch used to support video telecommunication; and (2) How does remote touch impact affective experience?

This paper is structured as follows: In Section 2, we present a background on the importance of interpersonal touch. Section 3 presents the related works and identifies what is currently lacking in the research area. Section 4 describes the scope of our research. Section 5 describes our touch system and experiment design, in addition to the methodology of our data analyses. In Section 6, we report the results obtained from the experiment. In Section 7, we discuss our findings, followed by some limitations and possible directions of future work.

## 2. The Importance of Interpersonal Touch

Interpersonal touch is an important channel for communication through which humans convey love, comfort, care, trust, and support. Communication through touch also supports human development [2] and well-being [3,4]. Touch can also strengthen the connection between people [5]. For example, skin-to-skin contact reassures infants that they are safe and protected, thus building the trust between child and parent [6]. Touch also creates a form of intimacy that can be better expressed without the use of language [7]. Fichten found that touch is more frequently used in dating situations than paralinguistic cues because touch is an impactful, yet more subtle, way of expressing interest [8].

The effects of touch are not merely psychological, but physiological as well. The lips and the fingertips are, according to McGlone, “more densely innervated and more cortically represented than other body sites” [9]. This is one reason why most exploratory and tactile behavior is done with the hands. Kissing has been found to improve perceived stress, relationship satisfaction, and total serum cholesterol. Chatel-Goldman also found that interpersonal affective physical touch elicited changes in physiological states within individuals—specifically, EDA “coupling” was increased [10]. Touch communication has also been shown to ameliorate responses such as heart rate, cortisol levels, and anxiety [11,12].

## 3. Related Works

Touch is one of the central forms of communicative experience and this can be communicated across a distance. To support remote touch-based communication, the concept of mediated touch has been explored. Mediated touch is a kind of touch that is delivered via haptic feedback technology, including tactile and kinesthetic devices [13] on areas such as the arm [14,15,16,17,18,19], lips [20,21,22], hand [23,24,25], and torso [26,27,28]. Mediated touch brings several benefits in human communication.

First, mediated touch enhances the quality of communication. This is not meant to replicate existing touch gestures, but rather to create a new vocabulary for social interaction. Chang’s ComTouch is a device that augments remote voice communication with touch [25]. They found that subjects employed three distinct types of touch (i.e., emphasis, turn-taking, and mimicry), demonstrating that a tactile channel can enhance voice communication. Singhal et al. presented Flex-N-Feel that communicates touch by vibrotactile gloves [24]. Their findings revealed that couples enjoyed their conversation more with the gloves, felt more emotionally connected, and experienced intimate moments together.

Second, mediated touch communication can be easily utilized in some difficult situations where other types of interactions cannot be executed. The Hug [26,29] is a haptic device that supports social and emotional communication in older adults who live far away from their families. With the Huggy Pajama [27], a parent or caretaker can send “hugs” to their child’s haptic jacket by squeezing a device that looks like a doll. Neither The Hug nor Huggy Pajama provided an evaluation of their systems.

Third, mediated touch provides opportunities to share non-verbal cues in intimate relationships. A wide range of experiments for mediated touch has been developed for dyads, especially close partners including romantic couples, parent-child, and friends. In this case, metaphoric touch events from real life have been utilized in communication. Hugs [26,27,28,29], kisses [20,21,22], pokes [30], handshakes [31], strokes [32], and messages [33] are popularly mediated through haptic devices.

Mediated touch is also considered a means for communicating specific emotions and can impact the recipient’s affect. In fact, simple forms of mediated touch can communicate a wide range of emotions [34,35,36]. Huisman’s tactile sleeve for social touch demonstrated that simple protracted touches (e.g., pressing and squeezing) may be sufficient to convey recognizable emotions through mediated touch [37]. However, these findings were not conclusive [38]. In Bailenson et al.’s study, participants were tasked to encode and decode six basic emotions (surprise, fear, disgust, anger, joy, and sadness) using a force-feedback joystick [31]. Results showed that all emotions were recognized at the above chance level. Smith and MacLean presented that dyads were able to communicate four different emotions (anger, delight, relax, unhappiness) using a knob and a set of haptically rendered models [39].

Mediated touch for successful affective communication depends heavily on context. In his survey of haptic technology for social touch, Huisman found that haptic technologies for social touch were “suitable” to communicate affect, but their effectiveness were highly dependent on context [40]. This finding was consistent in Eid’s paper on affective haptics—a term the author used to describe the field which “integrates ideas from affective computing, haptic technology, and user experience” [41]. Also consistent with Huisman’s findings, Askari found that, much like social touch, mediated touch is highly contextualized [42].

Prior work in mediated touch for affective communication has explored different contexts such as storytelling, visual feedback, relationship between interlocutors, and music. Wang and Quek showed that touch can impact the affective experience of an orally told story with a co-temporal touch stream [15] and the agreement of viewpoints between storyteller and listener [16]. Erk et al. explored the impact of mediated touch in remotely shared visual feedback and did not report any change in affective experience or trust towards the communication partner [43]. Chan et al. showed that a mediated touch stream experienced co-temporally with music impacts the affective experience music, but this depends on the relationship between the persons generating and receiving the touch [17].

## 4. Research Scope and Questions

With a few notable exceptions ([15,16,17,43]), most previous research on remote touch technology were design exercises with minimum evaluation or study on the affective impact. Although some works investigate the affective impact of mediated touch through subjective and sensor-based measures, very few explore both the psychological and physiological effects of remote touch in conjunction with speech. Our study utilizes several psychological and physiological instruments and sensor data to validate the effect of remote touch on real-time experience in emotionally rich conversations between committed, romantic partners.

Human language and interaction happen simultaneously on multiple time scales. We experience entire conversations with people, we move from topic to topic, we advance our conversation from utterance to utterance. Each unit of interaction moves the ‘joint project’ of the conversation and relationship forward. We framed this research to answer two research questions at different time scales in dyadic discourse situations.

1. How does remote touch support video telecommunication? (RQ1)
2. How does remote touch impact affective experience (physiological and psychological)? (RQ2)

We address the impact of our remote touch technology: (1) Across the entire telecon conversation: We applied a psychological questionnaire instrument to measure difference in positive and negative affect of the conversant before and after each discussion (with vs. without the use of touch devices); (2) Within the conversation, comparing utterances accompanied by touch against utterances without accompanying touch: We compared the distribution of Skin Conductance Response (SCR) peaks that fall on utterances with touch vs. utterances without touch; and (3) Within individual utterances: We measured: (a) the types of use that were appropriated by the touch devices for communication; and (b) the changes in electrodermal activity within utterances in the discourse. We hypothesize remote touch in video telecommunication will show similarities to natural touch in both use and affective response.

## 5. Materials and Methods

Each dyad was given one complete system to use in the experiment. The design of our system consists of a coupled input device and output device, as well as a recording software component. We utilized a similar hardware design and concept for our input and output devices as those presented by Wang and Quek [15].

Squeezes detected by the input device in each system controlled the kinesthetic touches generated by the output device worn by the other interlocutor. All touch exchanges, including their force intensities and durations, were facilitated and recorded by our software.

### 5.1. Hardware Prototype Design

**Figure 1:**
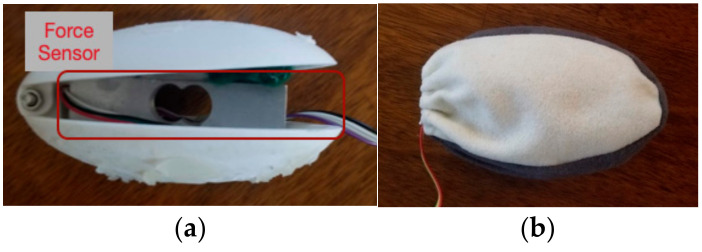
Prototype of Touch System’s Input Device. (**a**) Inside view of device; (**b**) Outside view of device.

The input device employs a hinged clamshell design with an incorporated force sensor. The two halves of the clamshell are 3D printed with PLA material. A 5 kg pressure sensor was mounted between the top and bottom halves of the device (see Figure 1a). This allowed us to measure the force when a user squeezed their device. The device was covered with a thin coat of silicone and enclosed in a soft fabric pouch to enhance tangible and tactile quality when squeezed (see Figure 1b). We fabricated the device this way to assist with measuring the force of a squeeze consistently while providing a more comfortable user experience.

Our input device design was derived through a course of user studies. We tested material and design alternatives in two preliminary studies. In the first preliminary study, we explored three designs: plain PLA model (Design A), plain PLA model inside fabric pouch (Design B), PLA model with thick silicone skin (Design C). Results revealed that the exerted and measured in Design A were the most consistent. Although Design B and C’s revealed more inconsistency in their measurements, the user experience scores were higher. To further improve the design of the device, we conducted an additional study to explore two designs that were informed by insights derived from the first study—a medium sized PLA model covered with a thin silicone skin and enclosed inside a fabric pouch (Design D) and a larger sized PLA model covered with a thin silicone skin and enclosed inside a fabric pouch (Design E). Compared to Designs A, B, C, and E, we discovered that Design D provided the most consistency in force readings and received one of the highest ratings in user experience.

**Figure 2:**
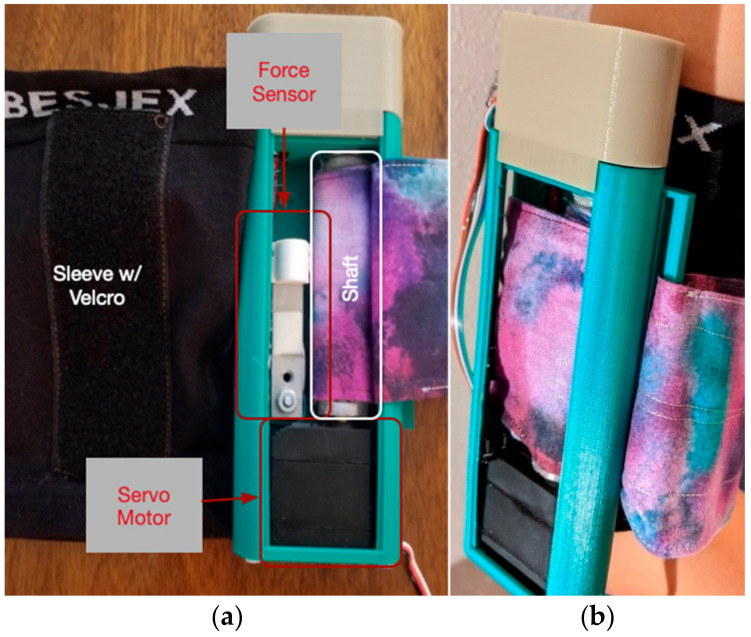
Prototype of touch system’s output device. (**a**) Inside view of device. (**b**) User wearing touch device on upper arm.

The output device consists of four main components: a servo motor-controlled rotating shaft that retracts and extends a fabric armband, a fabric armband whose fit to a user’s arm may be adjusted using a Velcro attachment, a force sensor, and an enclosure with Velcro tape along exterior of its back side. The subject wears a Velcro embedded compression sleeve on their upper arm while the Velcro on the device is attached to the exterior of the sleeve (see Figure 2 right). To ensure that the compression of the sleeve material did not obstruct the perception of the kinesthetic touches, we removed a section of the sleeve’s material to allow for the fabric armband to make direct contact with the subject’s bare (or close to bare) arm. The fabric armband is wrapped snugly around the subject’s arm, secured by Velcro. We chose the upper arm for our output device because it is a Non-vulnerable Body Part [44], and, as such, is a naturalistic area acceptable for different types of touch.

When the subject receives a touch, the sensor inside the output device measures the force of the squeeze between the armband and the subject’s arm. Attached at the end of the rotating shaft is a rotary encoder. This attachment serves two purposes: (1) it guides the shaft back to its original position after releasing the squeeze and (2) acts as a fail-safe to prevent over-squeezes that may result from any mechanical failure. A pilot study was conducted to characterize the dynamic range between different levels of squeezes that could be felt by users without discomfort.

### 5.2. Study Design

Our study is structured as a 2 (communication mode; no-touch, mediated-touch) × 2 (order; touch in first session or no touch in first session) counterbalanced, within-subjects experiment. Communication mode is a within-subject factor, and order is a between-subject factor. The duration of the experiment was approximately two hours long. For this study, we used the university’s bulk email service and recruited 22 heterosexual couples (44 total participants) who have been in their relationships for at least one year. The ages of the subjects ranged from 19 to 65.

At the start of the experiment, each subject was isolated in separate rooms. Biometric signals were recorded by the Empatica E4 wristband worn by the subjects. As recommended by the E4 user manual, the wristband was worn snuggly on the non-dominant hand [45] with the electrodes lined below the wrist and between the middle and ring fingers. An initial PANAS-X questionnaire was answered by each subject at this stage to measure a baseline in the responses. The experiment was divided into two sessions (one experiment condition each). Subjects watched a 15-min video at the beginning of each session. These videos presented relatable content that featured emotionally charged moments about work-life balance and romantic relationships. After watching the video, subjects participated in a perspective-sharing discussion with their partner through video tele-conferencing. The discussion was facilitated by semi-structured questions about the emotional highlights of the video. The remote touch devices were only given to use for the discussion in the with-touch condition session. Subjects were introduced to the operation of the devices prior to the discussion, but they were not instructed on how to use them in conversation. A two-minute practice session was provided for the couples to familiarize themselves with the experiment configuration (and as appropriate, with the touch technology) before the discussion.

PANAS-X responses were collected after each discussion session. Biometric data collection did not begin until the start of each discussion session, which was at least 30 min after the time the devices were worn. This allowed for ample time for the E4 to establish a baseline in the data. The E4 device monitored and recorded real-time physiological responses such as electrodermal activity (EDA), blood volume pulse, heart rate, skin temperature and accelerated wrist movements. The main focus of our experiment was the changes in arousal, found in EDA, which the E4 device samples at 4 Hz. To obtain the EDA measurements, the embedded electrodes in the E4 measure the electrical conductance in the wrist, which is affected by the amount of sweat the subject produces. For EDA, the E4 wristband records the timestamp and the conductance (measured in micro-Siemens).

### 5.3. Data Analysis Methodology

To examine how remote touches were used in the discussions, we identified the types of functions that were performed with the devices. We employed an open-coding approach (commonly used in qualitative analysis) to obtain an observer-based interpretation of the intention of each touch instance. This objective approach for measuring human experience is supported by grounded theory ([46,47,48,49]). Our analysis was divided into two rounds. We collected 2900 instances of touch transmissions that were coded for ‘intent’ using a multi-pass, multi-coder technique with inter-coder reliability checks and label resolution. In the first round, researchers independently and objectively labeled their observed purpose behind every sent touch, taking into account the surrounding context (e.g., speech, video, strength of touch, touch duration, etc.). We calculated an inter-rater reliability score of 0.88 using Cohen’s Kappa after the first round of open coding, which informs that researchers were in near perfect agreement with each other, despite coding independently from one another, thus validating the touch labels.

For the second round, researchers discussed their labeling to arrive at a “consensus label” for each touch. Consensus was determined in one of three ways. (1) If researchers independently reached the same label, a consensus label had been reached trivially. (2) In the event of a disagreement, researchers would have a discussion and choose a compromise label. (3) The two labels were “combined” if there was not a justifiable reason (as determined by the surrounding context of the touch) to choose one label over the other. Afterward, the full range of consensus labels (totaling 65 labels in all) were assigned into three categories: affective, non-affective and other. Affective touches aim to communicate or elicit an emotion (e.g., comforting, expressing happiness, expressing anger, etc.); non-affective touches aim to directly communicate a non-emotional message to the recipient (e.g., to request a speaking turn, to change a topic, to prompt a response, etc.); and other types of touches fit in neither non-affective nor affective touch categories (e.g., pragmatic touches to test the system). We present a glossary of each touch intention label in Appendix A.

To investigate the affective experience elicited by remote touch, we conducted a series of analyses on three different timescales (i.e., overall conversation, between utterances within the conversation, and within utterances) through several psychological and physiological instruments and sensor data.

First, we observed the affective impact of mediated touch in the overall conversations. Each subject’s PANAS-X (the Positive and Negative Affect Schedule-Expanded Form) [50] responses were recorded to measure their affective states at the beginning of the experiment and after each discussion session. The PANAS-X scale is a self-report questionnaire of distinguishable affective states within the dimensions of positive and negative emotional experiences. To assess the change in the subjects’ self-reported affective state, we compared the PANAS-X scores obtained from the no-touch (NT) condition to the responses from the with-touch (WT) condition.

Second, we investigated the overall physiological impact of mediated touch within the conversations. SCR was used to measure whether touch created a real-time response during the conversations. To understand how the speech (accompanied or not accompanied with touch) impacted the SCR in real-time, we segmented the discourse into timestamped utterances.

Discussion sessions were video recorded and transcribed. Our video recordings of the discussions were synchronized with the same master clock server used by the touch system’s recording software. All recorded touches that were sent and received bilaterally were timestamped using the master clock. This allowed researchers to more accurately observe the moments when touches were exchanged during the WT discussions. While reviewing the transcripts of the recorded video sessions, researchers divided the spoken discourse segments into purpose-level utterances (discourse segments with an overall purpose, as described by [51]). Our dataset of 19 couples (omitted 3 couples due to outliers) comprised audio-video data of 38 individuals, yielding 570 data minutes (15 min per person). Our purpose-based discourse coding produced a dataset of 25,662 utterances.

We used Ledalab (a widely used Matlab-based software for SCR analysis [52]) to analyze the raw EDA measurements to compare differences between significant skin responses elicited from utterances with and without touch. In Ledalab, we applied a low-pass filter to remove some noise and used the Continuous Decomposition Analysis method to retrieve the signal characteristics of the underlying sudomotor nerve activity. This method was also suggested to be more beneficial when performing analysis at unbiased scores of phasic and tonic activities in the EDA [53]. From this analysis, we obtained a record of the significant peaks (0.1 threshold) in the SCR amplitude. We obtained the temporal piece of when touches occur and compared it with the time of SCR peaks. We compared the SCR peaks across all utterances that do and do not contain touch.

Third, we conducted a fine-scale analysis on the changes in affective experience (measured by EDA data) within utterances. For this stage of our analysis, we examined how context (e.g., sentiment in speech, sex of partner, etc.) within each utterance may influence the changes in arousal. We conducted a two-way ANOVA test to investigate the interaction effects between the different types of context on the change in EDA response for each utterance (accompanied or not accompanied by touch).

To understand the emotional context within the conversations, we classified the types of sentiment in each utterance. Using FLAIR [54], we conducted sentiment analysis to label each utterance as having a “Positive,” “Negative,” or “Neutral” sentiment. We compared the results of the automated valence sentiment analysis with a small subset of our data that received manually coded valence scores. The inter-rater agreement between the manual and automated scores was 0.76 with Cohen’s Kappa analysis, which indicated that the FLAIR NLP labeling was acceptable for the rest of our dataset. Utterances that only contain backchanneling phrases such as “yeah,” “alright,” and “Mhmm” were excluded.

To measure the real-time changes in EDA, we used average EDA windows. These windows, constrained by a starting and ending EDA state, were calculated around each utterance to measure the change in response. The average of the EDA measurements that fall within a window of two seconds was considered a statistical representation of an EDA state. Our chosen window size is supported by [53], who found that the average duration of SCR responses was less than two seconds. The window placement to compute the starting EDA state of an utterance was as follows: If the participant was speaking, the window was placed right before the utterance. If the subject was listening to the utterance, the window was placed two seconds after the beginning of the utterance. This delay of two seconds was chosen because [55] showed that the galvanic skin responses are delayed 1–3 s after verbal stimulus. The ending EDA state of the utterances was computed with the window placed right after the end of the utterance for both, speaking and listening subjects.

## 6. Results

This study aims to investigate the affective impact of our remote touch technology on multiple timescales throughout the discussions. First, we present our findings on how the touch devices were used to support the dyadic discourse. Second, we present our findings on the changes in psychological and physiological affect at the same time-scale order in which we set forth our data analysis, as described in Section 5.3 (i.e., across the entire conversation, between utterances within the conversation, and within each utterance).

During the research process, two video files were corrupted, which have been removed from our analysis. Additionally, we removed one other couple from our dataset, due to it being a clear outlier. The ages of the remaining 19 couples ranged from 19 to 65 (mean age is 32, and median age is 26), and the lengths of their relationships ranged from one to 34 years.

### 6.1. How Is Remote Touch Used to Support Conversation (RQ1)?

We observed 65 different types of touch exchanges that were used in the WT discussions (see Appendix A for descriptions). These touches were classified into 3 broad categories: affective, non-affective, and other). Affective touches were intended to communicate emotions (e.g., loving, comforting, assurance, etc.). Non-affective touches aimed to communicate non-emotional messages (e.g., turn-taking, nudge, etc.). Other touches were used for purposes that were not meant for direct communication (e.g., testing the system, distracted, etc.).

Overall, we found that men received more touches than women (i.e., women sent more touches). While the majority of the received touches by women were affective touches, men mainly received touches intended for non-affective communication (see Table 1).

**Figure 3:**
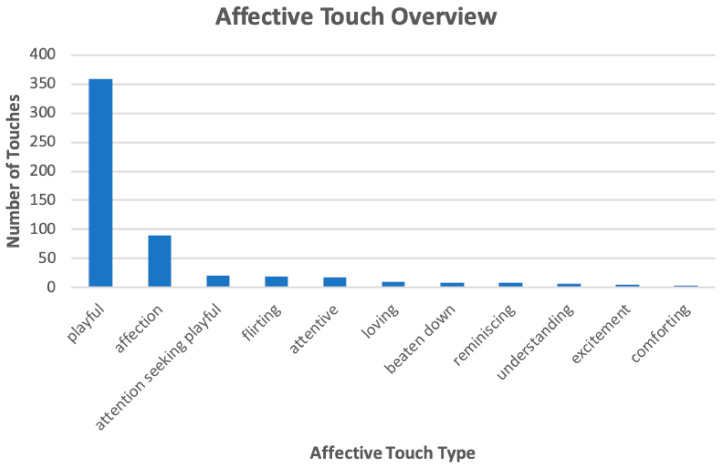
Distribution of Touches in Affective Sub-Categories.

**Figure 4:**
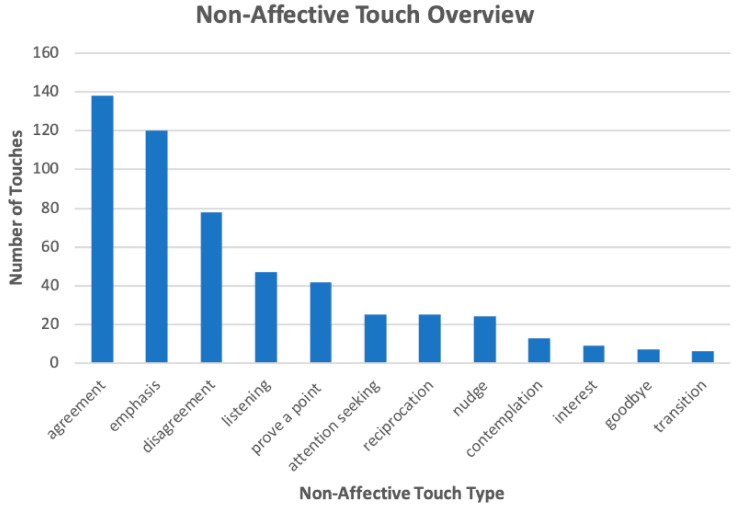
Distribution of Touches in Non-Affective Sub-Categories.

Figure 3 and Figure 4 present the distribution of the labeled touches that belong in the affective and non-affective categories, respectively. For readability purposes, we only present the types of touches with a count at least 5. The overall number of playful, agreement, and emphasis touches were greater than the number of touches in the other sub-categories. Playful touches made up 68% of all total affective touches, and agreement and emphasis touches made up 32% and 27% of all total non-affective touches, respectively.

These results suggest that users were able to quickly and naturally utilize the touch technology for a rich range of communicative, affective, and interactional (e.g., playfulness) intents.

Examples of Use

We present eight examples that demonstrate the range of use afforded by our system. For each case, we indicate the sender’s intention behind each touch (observed by researchers), the context behind the discourse (if necessary), and the speech between the dyads that accompanied the touch. All intentions were labeled based on the surrounding context of the observed conversation (e.g., tone of voice, body language, facial expressions, touch intensity, etc.). The overlap moments between speech and touch are underlined. Furthermore, if the touch happens within a pause in the speech, an ellipsis is underlined. Fictional names were used to protect the identity of the participant.

1. Intention: Comforting (affective)

Context: In a discussion on whether one should make a list of people to date, Woman (W) said:

W: “It’s just gonna be so straightforward and so blunt that it’s not OK for the other person to read … [received soft squeeze], but definitely for you to keep to yourself and just know.”

2. Intention: Affection (affective)

Context: Man (M) expressed opinion on whether there is a difference between a break and a breakup.

M: “I feel like breaks happen because they’re gonna have a breakup anyway [sent soft squeeze]. I’ve never heard of a healthy break.”

3. Intention: Anger (affective)

W: “There’s been times when you’ve come home from work, and we’ve had plans. And you’re like either ‘Oh, it’s too hot’ or ‘I’m too tired’. How do you think those answers make me feel when I’ve been home all day? ... [sent hard squeeze]”

4. Intention: Playful (affective)

Context: Man (M) is trying to make a point by comparing his relationship with the couple from the video clip.

M: “This is something [received medium squeeze] where we got it right [received medium squeeze] Stop this! ... [received medium squeeze]”

5. Intention: Affection (affective)

Context: Couple was having a discussion about whether Ross from the video was trying too hard to spend time with his girlfriend. Woman answered:

W: “Ross was trying way too hard. He should have just brought food and left it for her, and it would have been a thankful, thoughtful [sent medium squeeze] gesture.

6. Intention: Prove a point (non-affective)

Context: Woman (W) asked Man (M) if he would end their relationship to be with the girl of his dreams.

W: “What if Jennifer Aniston was like, “Yo, Carlos (actual name not used), I want to be with you.”

M: “No, because she might be a psycho.”

W: “But if you got to know her … [sent medium squeeze], and you were really friends for a while.”

7. Intention: Emphasis (non-affective)

Context: Man is about to explain why current discussion topic does not make sense to him

M: “I have no idea what that question even means. Here is the reason.”

W: “OK, you explain.”

M: “Number one … [sent soft squeeze]”

8. Intention: Agreement (non-affective)

Context: Man made a point that something inoffensive can easily sound offensive depending on how it is phrased. Woman (W) responded:

W: “You’re right, it’s not what they said, it’s how they said it. … [sent soft squeeze]”

These examples demonstrated that subjects used the touch devices in various parts of the conversation—e.g., while speaking, while listening to their partner speak, at the end of a sentence, at the end of a word, etc. This suggests that that subjects did not employ rigid ‘message passing’ with predefined protocols, contrary to previous works such as [37,38,39].

### 6.2. Psychological Affective Impact Across Conversations (RQ2)

From our PANAS-X data collected before and after the dialogic sessions, we found that the mediated touch impacted male and female subjects’ overall affect differently. In both men and women, the mediated touch created more surprise in their experience, but this effect was larger for women (women, t(18) = −3.63, *p* < 0.01; men, t(18) = −0.46, *p* = 0.65). The mediated touch also gave a larger positive impact on women (t(18) = −1.94, *p* < 0.10), but not for men (t(18) = 1.18, *p* = 0.25). Although the results did not cross the threshold for significance, the mediated touch appears to produce some positive impact in attentiveness for women (t(18) = −1.52, *p* = 0.14), but a negative impact in attentiveness for men (t(18) = 0.193, *p* = 0.07), compared to the no-touch condition.

### 6.3. Physiological Affective Impact between Purpose-Level Utterances (RQ2)

We investigated the overall physiological impact of mediated touch by comparing the significant SCR peaks in utterances coincident with the NT and WT conditions (see Table 2).

The difference approached significance between the means (t(33) = 1.94, *p* = 0.06) and the sums (t(33) = 1.99, *p* = 0.06). Mean and total SCR amplitudes were smaller in the WT condition and the NT condition, suggesting that mediated touch helped tame arousal.

### 6.4. Physiological Affective Impact within Purpose-Level Utterances (RQ2)

As suggested by previous literature, context shapes how mediated touch is experienced ([15,16,17,40,41,42]). We conducted a two-way ANOVA test to investigate the interaction effects between different types of factors (i.e., context) such as sex of the partners, sentiment of the utterances, and type of remote touch (affective, non-affective, or other) on the change in EDA measurements in each utterance. Table 3 highlights the significant main effects on change in arousal when all types of touches (affective, non-affective, and other) are considered.

For each significant interaction between effects on the change in EDA measurements, we conducted a deeper analysis and investigated its contributing simple effects.

We found that affective touches elicited a stronger reduction in arousal than non-affective (F(1, 1191) = 61.46, *p* < 0.001) and other (F(1, 617) = 12.92, *p* < 0.001) touches. However, we did not find significance between the effects of non-affective and other touches on arousal change at the *p* = 0.10 level.

**Figure 5:**
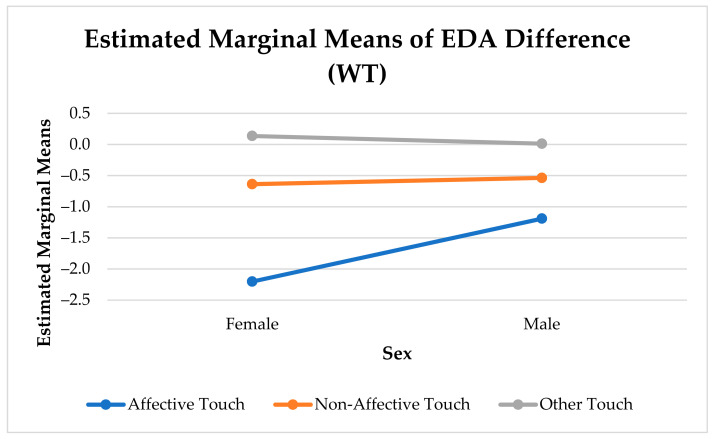
Sex Differences in Physiological Arousal Change Elicited by Touch.

Figure 5 shows the significant interaction between the effects of sex and type of touch on the change in arousal. There was a difference in arousal change between affective and non-affective touches (F(1, 545) = 41.14, *p* < 0.001), and affective and other touches (F(1, 296) = 11.54, *p* < 0.001) for females. In males, we only found a significant difference in arousal change between affective and non-affective touches (F(1, 644) = 20.21, *p* < 0.001). While affective and non-affective touches elicit a notable reduction in arousal, other touches barely elicit any arousal change.

When sentiment of the utterances was included in our analysis, we found no significant difference in arousal change between utterances with or without touch for men, irrespective of sentiment (Table 4).

**Figure 6:**
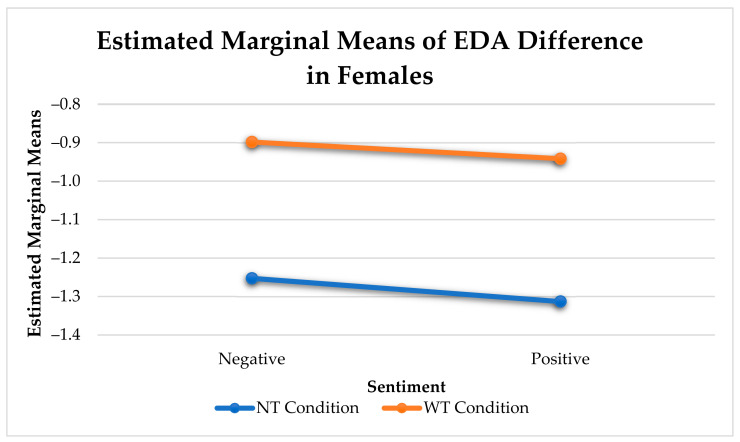
Change in Physiological Arousal Elicited by Sentiment and Touch for Females.

In females, there was a significant difference between utterances with touch and without touch for negative sentiment (see Figure 6).

Overall, we did not find a significance difference between the arousal change in negative and positive utterances, irrespective of touch condition (Table 5). We only found a significant difference in the arousal change elicited by negative and positive NT utterances for males (F(1, 9497) = 12.86, *p* < 0.001).

**Figure 7:**
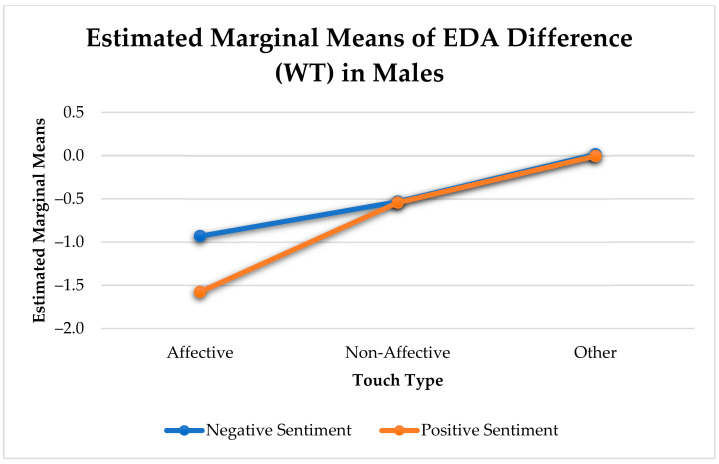
Physiological Arousal Change Elicited by Touch for Positive and Negative Sentiment in Males.

As shown in Table 6, there was a significant arousal change between negative and positive utterances that contain an affective touch in men only (F(1, 650) = 9.36, *p* < 0.005). Non-affective and other touches did not show any effect (Figure 7). We did not find any significant effects for women.

Our findings in this subsection revealed that when all categories of touch are considered, there were few significant findings. Other touches seemed to elicit very little change in arousal compared to affective and non-affective touches, which may have impacted the overall results. To further investigate, we dove deeper in this analysis and examined the significant interaction effects that occur: (a) between affective and non-affective touches (see Section 6.4.1), (b) affective touches only (see Section 6.4.2), (c) non-affective touches only (see Section 6.4.3), and (d) other touches only (see Section 6.4.4).

#### 6.4.1. Physiological Affective Impact Elicited by Affective and Non-Affective Touches

We conducted a two-way ANOVA test on the effects of factors such as the sex of the partners, the sentiment of the utterances, and type of remote touch (affective or non-affective) on the change in arousal in each utterance. Significant findings in main effects are presented in Table 7.

For each main interaction between effects that had a significant impact on the change in arousal, we conducted a deeper analysis and investigated its contributing simple effects.

**Figure 8:**
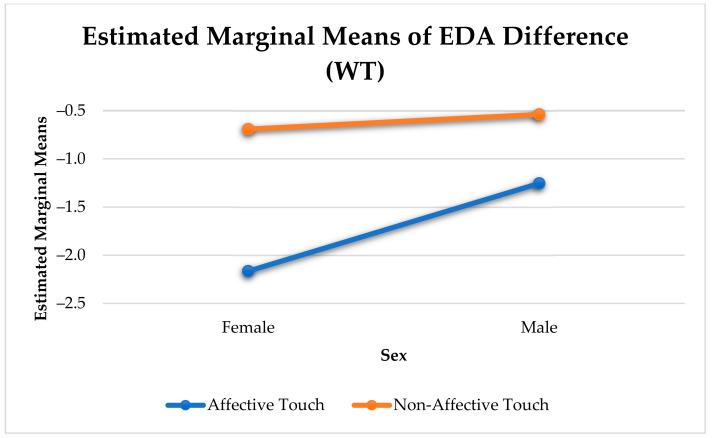
Sex Differences in Physiological Arousal Change Elicited by Affective and Non-affective Touch.

Females experienced a significantly stronger reduction in arousal than males (F(1, 13295) = 160.53, *p* < 0.001). Similar to the analysis with all three categories of touches, there was a significant difference in arousal change between affective and non-affective touches for males (F(1, 644) = 20.21, *p* < 0.001) and females (F(1, 545) = 41.14, *p* < 0.001) (see Figure 8).

When we included the sentiment of the utterances in our analysis, we found a significant difference between response from positive and negative utterances that contained affective or non-affective touch for males, but not females (see Table 8).

**Figure 9:**
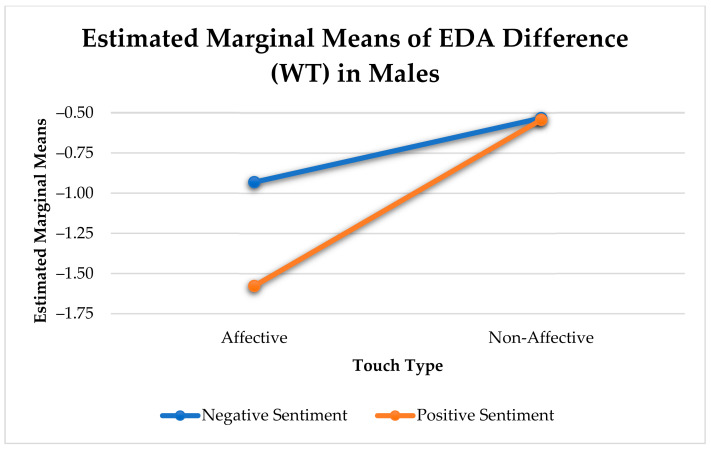
Physiological Arousal Change Elicited by Touches with Negative or Positive Utterances in Males.

As previously mentioned, utterances with positive sentiment that received affective touches elicited significantly greater reduction in arousal change than affective or non-affective touch utterances with negative sentiment for males. When both affective and non-affective touches are considered as a collective, we noticed a significant difference between the arousal change in negative and positive arousal. However, this change was largely influenced by affective touches (see Figure 9).

Our findings showed that communicative remote touches elicited a real-time response in EDA, and we found a significant difference between the EDA change elicited by affective and non-affective touches. Though both non-affective and affective touches reduced arousal, affective touches created a stronger ameliorating effect.

#### 6.4.2. Physiological Affective Impact Elicited by Affective Touch

We conducted a two-way ANOVA test on factors such as the sex of the partners, the sentiment of the utterances, and touch condition on the change in arousal in each utterance. Only affective touches were considered (for WT utterances). Table 9 highlights the significant main effects.

**Figure 10:**
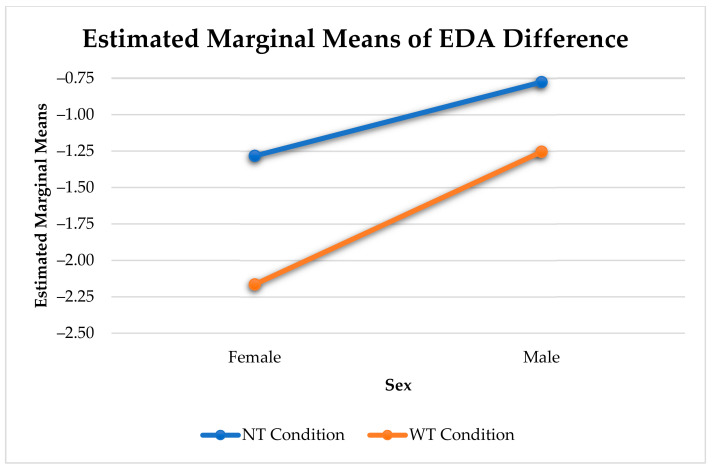
Sex Differences in Physiological Arousal Change Elicited by Utterances with or Without Affective Touches.

Consistent with previous analysis, females experienced a greater reduction in arousal change than men (F(1, 582) = 15.19, *p* < 0.001). Affective touch utterances elicited a greater reduction in arousal change than utterances without touch (F(1, 6641) = 50.92, *p* < 0.001). This significant difference remains when we compare the same effect between sexes (Figure 10).

**Figure 11:**
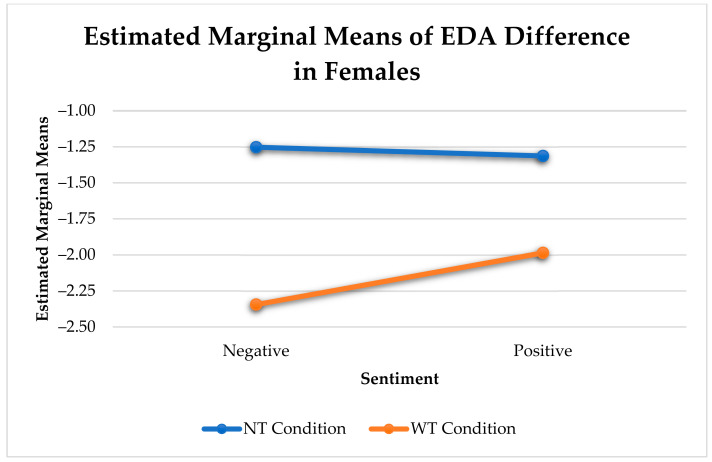
Differences in Physiological Arousal Change Elicited by Utterances with or Without Affective Touches for Positive and Negative Sentiment in Females.

Table 10 presents the significant interaction between the effects of sex, sentiment, and touch condition (affective touch vs. no touch) on the change in arousal. Affective touches elicited a greater reduction in arousal compared to utterances without touch during either positive (F(1, 9492) = 6.73, *p* < 0.01) or negative utterances (F(1, 9492) = 2697, *p* < 0.001) for females (see Figure 11).

**Figure 12:**
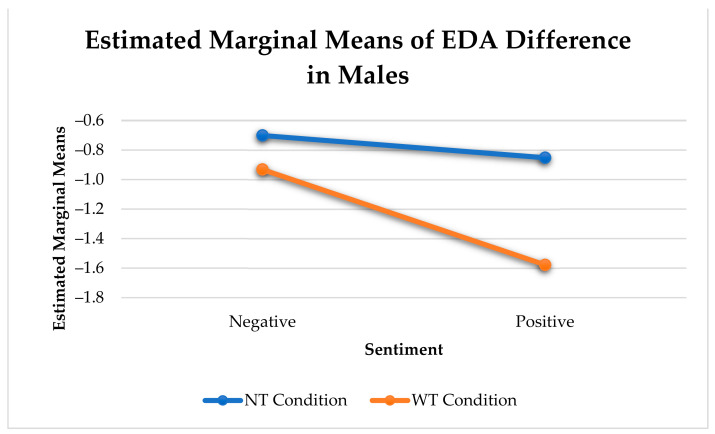
Differences in Physiological Arousal Change Elicited by Utterances with or Without Affective Touches for Positive and Negative Sentiment in Males.

Only positive utterances with affective touches elicited a significantly greater reduction in arousal than NT negative utterances for males (F(1, 9806) = 15.653, *p* < 0.001) (See Figure 12).

#### 6.4.3. Physiological Affective Impact Elicited by Non-Affective Touch

We conducted a two-way ANOVA test on factors such as the sex of the partners, the sentiment of the utterances, and touch condition on the change in arousal in each utterance. Only non-affective touches were (for WT utterances). Table 11 highlights the significant main effects.

**Figure 13:**
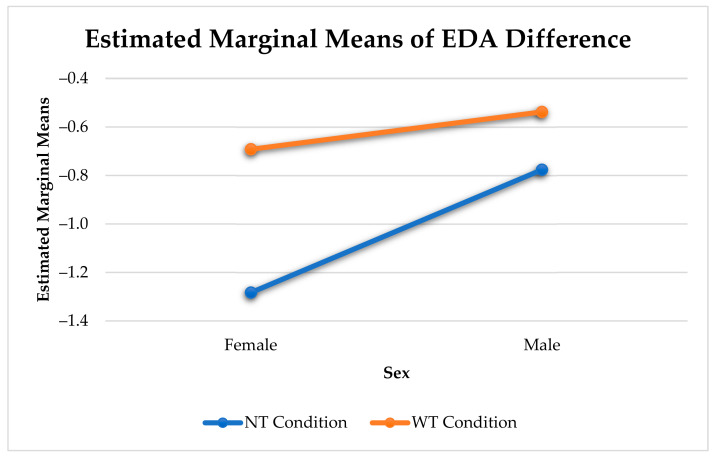
Sex Differences in Physiological Arousal Change Elicited by Utterances with or Without Non-Affective Touches.

Non-affective touch utterances elicited a greater reduction in arousal change than utterances without touch (F(1, 19323) = 17.54, *p* < 0.001). As shown in Figure 13, this effect holds between sexes (females: F(1, 9498) = 11.86, *p* < 0.001; males: F(1, 9830) = 4.32, *p* < 0.05). Although non-affective touches elicited real-time changes in arousal, these responses were not influenced by the type of emotional context in the conversations.

#### 6.4.4. Physiological Affective Impact Elicited by Other Touch

We did not find significant effects for other touches on the change in arousal. This suggests that touches that were not used for direct communication elicited minimal change in physiological response.

## 7. Discussion

Our touch devices were used primarily in both discourse regulation and affective exchange (i.e., non-affective and affective touches). As shown in Figure 3 and Figure 4, our device affords a rich range of use. Users were able to naturally (i.e., without any instruction) utilize the technology for a rich range of communicative, affective, and interactional (e.g., playfulness) intents spontaneously throughout their conversations. This is contrary to most prior work on mediated touch that employed rigid ‘message passing’ with predefined protocols such as [37,38,39]. This finding suggests that remote touches were used similarly to how we use nonverbal cues in natural touch [8]. Just as the use of non-verbal cues during in-person interactions depend on a person’s particular communication preference, the use of touch during tele-communication also varies. We establish that remote touch, like natural touch and other forms of non-verbal communication, is used as a communication channel to convey information beyond that passed by prosody or specific wording. The use of a novel device to provide touch remotely does not unduly inhibit communication or disrupt the flow of conversation. This shows the tremendous potential of the technology as a channel for interpersonal distance communication for affective and conversation regulation purposes.

Our findings revealed different levels of affective impact and touch use that occurred at different time scales of the conversation.

At the conversation level, PANAS-X responses revealed a small glimpse into the affective impact that mediated touch created. Overall, women experienced a stronger positive effect in their affective experience and men experienced less attentiveness when remote touches were used during the discussion. Although our generalizations are based on this single study with only 19 couples, our findings still uncover this difference of the technology across sexes. We suspect that mediated touch created different types of impact between sexes because women have a higher preference for mediated communication, as suggested by [5,56,57,58]. For instance, women had a stronger preference for text messaging, social networking, video chat, and phone calls compared to men. Women also spent more time communicating with friends via mediated communication. Although our results suggest that there is a differential effect of the technology with the sex of the participant, their age could also have played a factor [59]. Furthermore, the participants are being exposed to the technology for the first time. Literature suggests that men have a preference for exploring the affordances of new technology while women have a preference for the social utility [60]. It is not clear how more familiar use of the novel technology may relate to the different sexes. Hence, further research is needed to determine the extent and manner sex differences affect the use of remote touch.

At a deeper level into the conversations, our findings revealed changes in physiological affective experience (measured by SCR peaks and average EDA windows) when remote touches were received in conjunction with speech. We found that subjects experienced a reduction in their SCR response when mediated touch was received. Our findings on the general reduction in arousal response is consistent with how affective natural touch typically serves a calming function in stressful situations [11,12]. Though the SCR measurements were sparse, it still provided a broad understanding that the remote touch indeed created a contemporary impact during the conversations. This suggests that remote touch may be used in situations to reduce anxiety and decrease cortisol levels during dyadic communications.

Similar to natural touch, mediated touch requires additional context [15,16,17]. We examined how the context surrounding the remote touch (e.g., emotional context of each utterance and intent of touch) within each utterance influenced the changes in EDA measurements. When the context of the utterances (i.e., positive or negative sentiment) is considered, only affective touches created a difference in arousal reduction. As discussed earlier, these findings align with research that show that natural touch ameliorates arousal [11,12]. It also makes sense that the emotional context of the discourse did not influence EDA response from non-affective touches—non-affective touches are more for instrumental use rather than affective conveyance. These findings demonstrate the importance of context when measuring the impact of remote touch. When the touch is observed to have an affective intent, emotive experience is impacted. This is consistent with our observation of the rich and multivariate use of remote touch interaction. To further investigate the influence of emotional context in remote touch conversations, future studies may explore other variations of emotional context in the utterances (e.g., affective state). We also found that touches labeled as “other” elicited minimal change in arousal whereas affective and non-affective touches elicited significant arousal reductions. This shows that the emotive impact is not purely physiological, brought about by the fact of remote touch interaction alone. Our findings suggest that EDA, when used as averaging windows, is an effective measure and correlates well at the significant level with human understanding of what the discourse and affective purpose of the mediated touch is. This opens the opportunity for sensor technology to more effectively provide feedback in applications that require real-time affective mediation. Such examples include new technology that may utilize biofeedback for counseling situations or to emotionally support individuals at risk for loneliness, stress, and anxiety.

We note that including other data (e.g., amplitude, duration, and time overlap) in our analysis of the squeezes with dialog and utterance segments may be useful. This may, in fact, provide a ‘super-segmental analysis’ of the speech beyond individual utterances. This is, however, beyond the scope of this work--we are focused on the before-and-after affective experience relating to individual utterances with respect to touch intentionality (e.g., affective vs. communicative vs. other touch). Our research is intended as one such seminal point of research in what we hope will innervate new directions of research involving a broader range of sensor data.

## 8. Limitations and Directions for Future Work

One limitation of this study is the relatively small number of subjects who participated in the experiment. Research of the kind presented involves intensive coding and micro-analysis of hundreds of minutes of discourse, producing tens of thousands of multimodal discourse elements that are then further analyzed (verbal coding, classification, etc.). Three classic studies by Chi, Roscoe, and Markman, (all Google citation counts are above 200) employed about 11–13 dyads [61,62,63]. Perhaps with a larger dataset, other interesting findings would have also crossed the significance threshold. However, even with the small number of subjects, we still found significance in our results (with averaging window analysis).

However, given the variability in how individuals may employ remote touch technology, we anticipate other studies that may explore differences between remote touch use across cultures, ages, sexual-orientations, and in greater numbers. This brings to the second and third limitations of our research.

The second limitation of our study is the broad age range in our subjects. While there is research that shows that natural touch in older committed couples differ somewhat from touch between younger couples (In heterosexual couples, female to male touch are more frequent in older couples, while male-initiated touch are more prevalent in younger couples) [59], there is no evidence of different affective and communicative use of touch. We note that given our recruitment through a university listserv, the median age of our participants is 26, with the only two participants under 20 years of age (both 19). Beside students, university listservs also include staff and faculty of which we have 4 between 40 and 50 years, 3 between 50 and 60, and 4 between 60 and 65. This age range did not hinder us from finding significance in our data is interesting, indicating that the use of our touch technology is quite consistent across ages. In future studies, we may set age requirements to see if there are systematic differences across age groups.

The third limitation of our study is that the participants represent a single sexual orientation in romantic couples. Our goal was to use participants for whom interpersonal touch would not be strange (i.e., between romantic partners)—and add a confound to our study. When recruiting for subjects, we requested for any couples in committed relationships of at least one year to participate in our study. All couples who volunteered happened to be heterosexual. Future studies that compare homosexual couples may provide a more comprehensive picture on the impact of mediated touch between romantic partners.

A fourth limitation of our study is that we only observed the impact of kinesthetic squeezes to the upper arm. Other types of touch (e.g., cutaneous touch, touch with temperature, etc.) used in dyadic video telecommunication on various areas of the body may suggest different results. This study is but a starting point for such broader exploration.

## 9. Conclusions

We developed a remote touch system that enriches the affective and communicative interaction between interlocutors in video telecommunication. In this study, committed partners used a pair of coupled input and output devices to send real-time kinesthetic squeezes to one another in semi-structured discussions about emotionally charged topics. Psychological and physiological instruments were employed to measure the affective impact of co-verbal touch in their conversations. The key contribution of our work is that most prior work with haptic touch devices stopped short of studying their effectiveness in dyadic discourse situations, how such technology impacts affective experience, and how these experiences may be detected and measured through EDA. When given the touch devices, couples were able to spontaneously appropriate remote touch into their conversations, without any specific instructions on how or when to do so. Just as the use of other non-verbal cues like gestures during in-person interactions depend on a person’s particular communication preference, we expect the use of touch during tele-communication to also vary. What is clear from our study, however, is that this variation exists within a landscape of rich interaction between the dyads. Without instruction on how to operate the devices, subjects appropriated 65 different types of remote touch use in their discussions to convey affective and other communicative intents. These devices were also utilized spontaneously, the opposite of the rigid message passing protocols employed by most previous work. We establish that remote touch, like in-person touch, relies on context to support communication and convey information beyond, but in conjunction with, that passed by prosody or specific wording. Remote touch experienced with additional context (e.g., emotional topics of conversation or purpose of touch) can influence one’s affective experience differently. The use of a novel device to provide touch remotely does not unduly inhibit communication or disrupt the flow of conversation, but rather provides conversational support, and when the support is affective, it produces a measurable change in skin conductance response. Our findings provide a starting point for new technologies with real-time feedback mechanisms for a range of communicative and affective support.

## Figures and Tables

**Table 1:** Descriptive Overview of Total Received Touches (grouped by Intention Type).

Sex	Touch Type	Sum	Mean	Std. Dev	Min	Max
Female	Affective	276	16.2	14.1	1	40
	Non-Affective	218	12.8	13.7	0	60
	Other	94	5.5	13.4	0	56
Male	Affective	288	16.9	19.3	2	63
	Non-Affective	331	19.5	22.9	2	93
	Other	143	8.41	9.0	1	39
Overall	Affective	564	16.6	16.6	1	63
	Non-Affective	549	16.2	18.9	0	93
	Other	237	7.0	11.3	0	56

**Table 2:** Overall SCR Amplitude for NT and WT Conditions.

Condition		SCR_Amp Sum	SCR_Frequency	SCR_Amp Mean
NT	Mean	18.3	83.9	0.2
	N	34	34	34
	Standard Dev.	29.9	143.8	0.3
WT	Mean	10.6	51.5	0.16
	N	34	34	34
	Standard Dev.	14.8	28.4	0.17

**Table 3:** Significant main effects on change in physiological arousal from remote touch (all types).

Effect	df	F	*p*-Value
Touch Type	2	32.13	<0.001
Sex × Touch Type	2	3.76	0.02
Sex × TouchType × Sentiment	2	2.78	0.06

**Table 4:** Sex Comparison Between Physiological Arousal Change in Utterances (NT vs. WT) by Sentiment Type.

Sex	Sentiment	Condition (I)	Condition (J)	Mean Difference (I-J)	*p*-Value
Female	Negative	NT	WT	−0.35	0.09
	Positive	NT	WT	−0.37	0.28
Male	Negative	NT	WT	−0.22	0.43
	Positive	NT	WT	−0.14	0.86

**Table 5:** Sex Comparison Between Physiological Arousal Change in Positive and Negative Utterances by Touch Condition.

Sex	Condition	Sentiment (I)	Sentiment (J)	Mean Difference (I-J)	*p*-Value
Female	NT	Negative	Positive	0.06	0.22
	WT	Negative	Positive	0.04	0.91
Male	NT	Negative	Positive	0.15	0.002
	WT	Negative	Positive	0.23	0.79

**Table 6:** Sex Comparison Between Physiological Arousal Change in Positive and Negative Utterances by Touch.

Sex	Touch Type	Sentiment (I)	Sentiment (J)	Mean Difference (I-J)	*p*-Value
Female	Affective	Negative	Positive	0.30	0.30
	Non-Affective	Negative	Positive	0.36	0.37
	Other	Negative	Positive	0.90	0.90
Male	Affective	Negative	Positive	.211	0.002
	Non-Affective	Negative	Positive	0.21	0.96
	Other	Negative	Positive	1.93	0.99

**Table 7:** Significant main effects on change in physiological arousal from affective or non-affective mediated touch.

Effect	df	F	*p*-Value
Sex	1	14.1	<0.001
Touch Type	1	59.61	<0.001
Sex × Touch Type	1	7.13	0.008
Sex × Sentiment × Touch Type	1	5.41	0.02

**Table 8:** Sex Comparison Between Physiological Arousal Change in Positive and Negative Utterances by Touch Condition.

Sex	Condition	Sentiment (I)	Sentiment (J)	Mean Difference (I-J)	*p*-Value
Female	NT	Negative	Positive	0.06	0.22
	WT	Negative	Positive	−0.02	0.94
Male	NT	Negative	Positive	0.15	0.002
	WT	Negative	Positive	0.33	0.08

**Table 9:** Significant Main Effects on Change in Physiological Arousal. Only affective touches were considered.

Effect	df	F	*p*-Value
Sex	1	48.97	<0.001
Sex × Condition	1	3.98	0.046
Sex × Condition × Sentiment	3	2.71	0.04

**Table 10:** Sex Comparison Between Physiological Arousal Change in Positive and Negative Utterances by Touch Condition.

Sex	Condition	Sentiment (I)	Sentiment (J)	Mean Difference (I-J)	*p*-Value
Female	Negative	NT	WT	1.09	<0.001
	Positive	NT	WT	0.67	0.003
Male	Negative	NT	WT	0.23	0.19
	Positive	NT	WT	0.73	<0.001

**Table 11:** Significant Effects on Change in Physiological Arousal. Only non-affective touches were considered.

Effect	df	F	*p*-Value
Sex	1	10.59	0.001
Condition	1	15.76	<0.001
Sex × Condition	1	3.04	0.081

## Data Availability

Data sharing not applicable.

## Appendix A

In this section, we present a brief definition for each label we used to open code the types of intentions of each sent touch. These terms are described with respect to the conversations between dyads during the study.

### Appendix A.1. Affective Touch Intentions

The touches placed in the affective category are described as follows:

1. Affection: Conveying love or fond attachment
2. Affection Assurance: Showing affection while providing assurance
3. Affection Reciprocation: Showing affection while reciprocating
4. Agreement Assurance: Providing assurance while in agreement
5. Agreement Understanding: Showing understanding while in agreement
6. Anger: Slight hostility aroused by wrath
7. Appreciation: Thankful recognition
8. Assurance: To give confidence or surety
9. Attention Seeking Playful: Showing playfulness while seeking attention
10. Attentive: Paying close attention in a thoughtful or loving way
11. Attentive Assurance: Providing assurance while remaining attentive
12. Attentive Loving: Expressing love while remaining attentive
13. Beaten down: In a continuous state of happiness or mortification
14. Comforting: To afford consolation or solace
15. Comforting Affection: Comforting while expressing affection
16. Comforting Assurance: Comforting while providing assurance
17. Comforting Beaten Down: Comforting while feeling beaten down
18. Contemplation Assurance: Providing assurance while contemplating
19. Disagreement Understanding: Express understanding while in disagreement
20. Excitement: Expression of great enthusiasm
21. Flirting: To act amorous in a playful way
22. Flirting Loving: Flirting while expressing love
23. Frustration Disagreement: Feelings of frustration while in disagreement
24. Goodbye Affection: An affectionate goodbye
25. Laughing: Audible laughter
26. Loving: Conveying warm affection
27. Loving Agreement: An agreement while expressing feelings of love
28. Playful: Having fun or jesting
29. Playful Affection: Showing affection while expressing playfulness
30. Playful Agreement: An agreement while expressing playfulness
31. Playful Agreement Assurance: Providing assurance while expressing playful agreement
32. Playful Anger: Showing anger while expressing playfulness
33. Playful Flirty: Flirting while also expressing a deeper playfulness
34. Playful Inquiry: To ask a question while expressing playfulness
35. Playful Other: Playful exchanges at the end of the discussion session with little to no context
36. Playful Prove a Point: To prove a point while expressing playfulness
37. Playful Reciprocation: To reciprocate to a touch while expressing playfulness
38. Playful Threatening: Threatening partner in a playful manner
39. Reminiscing: Fondly recall past experiences or events
40. Surprised: To enter in a state of shock
41. Understanding: An expression of empathy
42. Upset: Expressing displeasure or emotional disturbance

### Appendix A.2. Non-Affective Touch Intentions

The touch intentions placed in the non-affective category are described as follows:

43. Agreement: Convey consensus or aligned perspectives
44. Agreement Attentive: An agreement while remaining attentive
45. Attention Seeking: Eliciting attention, either towards self or discussion topic
46. Attention Seeking Nudge: Elicit a response from partner while also seeking attention
47. Be Serious Attention Seeking: Seeking attention and telling partner to be serious
48. Clarification Attention Seeking: Seeking attention while requesting for clarification
49. Contemplation: Entering into deep thought
50. Disagreement: Lack of approval or consensus due to unaligned perspectives
51. Emphasis: Accenting words to signify importance
52. Emphasis Agreement: Emphasizing an agreement
53. Goodbye: Say goodbye
54. Inquiry: Ask a question
55. Interest: Expressing curiosity in partner’s perspective
56. Listening: Paying attention to what partner is saying
57. Nervous: Expressing apprehension
58. Nudge: Soliciting a response
59. Prove a Point: Asserting reasons for disagreement
60. Reciprocation: An immediate response to a received squeeze
61. Reciprocation Agreement: Reciprocation while in agreement
62. Transition: Indication to move on to the next topic of the prompted discussion

### Appendix A.3. Other Touch Intentions

The touches placed in the other category are described as follows:

63. Distracted: Unintentionally shifted focus away from the teleconference
64. Playing: User is playing with the device
65. Testing: Testing if the device is working
